# The role and mechanism of TCM in the prevention and treatment of infectious diseases

**DOI:** 10.3389/fmicb.2023.1286364

**Published:** 2023-11-14

**Authors:** Qifei Zou, Yitong Chen, Huanxin Qin, Rui Tang, Taojian Han, Ziyi Guo, Juanjuan Zhao, Delin Xu

**Affiliations:** ^1^Department of Medical Instrumental Analysis, Zunyi Medical University, Zunyi, Guizhou, China; ^2^Department of Immunology, Zunyi Medical University, Zunyi, Guizhou, China

**Keywords:** traditional Chinese medicine, infectious diseases, mechanism, prevention and treatment, active component

## Abstract

The constant presence of infectious diseases poses an everlasting threat to the entire world. In recent years, there has been an increased attention toward the application of traditional Chinese medicine (TCM) in the treatment of emerging infectious diseases, as it has played a significant role. The aim of this article is to provide a concise overview of the roles and mechanisms of TCM in treating infectious diseases. TCM possesses the ability to modulate relevant factors, impede signaling pathways, and inhibit microbial growth, thereby exhibiting potent antiviral, antibacterial, and anti-inflammatory effects that demonstrate remarkable efficacy against viral and bacterial infections. This article concludes that the comprehensive regulatory features of Chinese herbal medicines, with their various components, targets, and pathways, result in synergistic effects. The significance of Chinese herbal medicines in the context of infectious diseases should not be underestimated; however, it is crucial to also acknowledge their underutilization. This paper presents constructive suggestions regarding the challenges and opportunities faced by Chinese medicines. Particularly, it emphasizes the effectiveness and characteristics of Chinese medicines in the treatment of infectious diseases, specifying how these medicines’ active substances can be utilized to target infectious diseases. This perspective is advantageous in facilitating researchers’ pharmacological studies on Chinese medicines, focusing on the specific points of action. The mechanism of action of Chinese herbal medicines in the treatment of infectious diseases is comprehensively elucidated in this paper, providing compelling evidence for the superior treatment of infectious diseases through Chinese medicine. This information is favorable for advancing the development of TCM and its potential applications in the field of infectious diseases.

## 1. Introduction

Infectious diseases, such as respiratory diseases, vector-borne diseases, and sexually transmitted diseases, are the leading causes of illness and death globally (Lou, 2020). Despite the progress made in preventing and controlling these diseases through traditional Chinese medicine (TCM), they remain a persistent threat (Chowell and Rothenberg, 2018; Johnson et al., 2023). The outbreak of COVID-19 in Wuhan, China in November 2019 (Baker et al., 2022; Oke et al., 2022) resulted in a rapid worldwide spread of a contagious disease caused by SARS-CoV-2 (Safiabadi Tali et al., 2021). This global pandemic has resulted in hundreds of thousands of deaths, highlighting the devastating impact of infectious diseases and emphasizing the need for prevention and treatment measures (Shi et al., 2020; Blutt and Estes, 2022).

**Figure 1:**
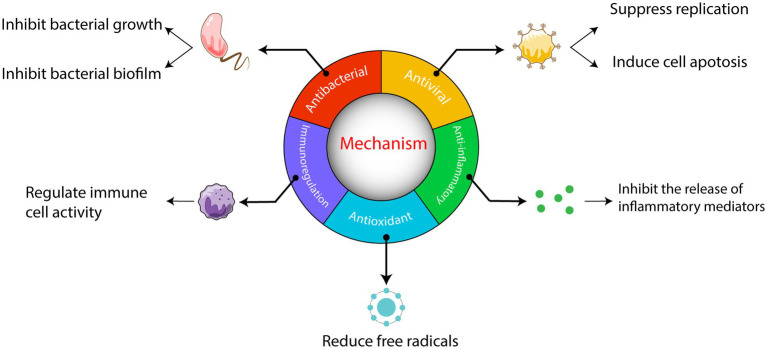
Mechanism outline of TCM on treating infectious diseases.

Historically, China has experienced numerous devastating infectious diseases from 243 BC to 1949. TCM has achieved positive outcomes in treating these diseases by effectively controlling outbreaks through the use of TCM by the public (Ni et al., 2020). In the current pandemic, multiple clinical practices have shown the significant role of TCM in treating COVID-19, with reliable reports provided to the scientific community (Lee et al., 2021). Chinese herbal medicine, as an integral part of Chinese culture, possesses unique advantages and plays a vital role in preventing and treating infectious diseases (Zhao et al., 2021). It can reduce the severity of diseases, aid in clinical recovery, and demonstrate better efficacy with fewer side effects. This has generated increased interest in the potential of herbal medicine in treating infectious diseases (Ni et al., 2020; Yang et al., 2020). Numerous TCM have proven effective in the clinical treatment of various infectious diseases (Wang et al., 2021). The mechanisms by which Chinese herbal medicine treats these diseases primarily involve antiviral, antibacterial, and anti-inflammatory effects. These effects are primarily attributed to active substances found in alkaloids, flavonoids, polysaccharides, saponins, tannins, and polyphenols. The comprehensive nature of TCM, with its multiple components, targets, and pathways, contributes to synergistic effects. Existing reviews mostly focus on describing the mechanisms of different Chinese herbal medicines, with only a few providing systematic reviews of multiple mechanisms. This article aims to explain the various mechanisms of Chinese herbal medicines in treating infectious diseases and discuss the challenges, prospects, and potential modernization of Chinese herbal medicine (Figure 1). Its objective is to serve as a reference for the advancement of traditional Chinese medicine.

In this article, we used keywords such as “traditional Chinese medicine,” “infectious diseases,” “traditional Chinese medicine AND antibacterial mechanisms AND infectious diseases,” “traditional Chinese medicine AND immune regulation AND infectious diseases,” and “traditional Chinese medicine AND antiviral mechanisms AND infectious diseases” to search for literature on the treatment of infectious diseases by traditional Chinese medicine in the past 20 years on PubMed, CNKI, and ScienceDirect. The different mechanisms of traditional Chinese medicine in treating infectious diseases were systematically studied.

## 2. Herbal medicine used in the treatment of infectious diseases

### 2.1. Significant role of major Chinese herbal medicines in infectious diseases

There is abundant documentation and experimental evidence that proves the extensive use of herbal medicines in the treatment of infectious diseases. These medicines play a vital role in combating various diseases and maintaining human health. Many drugs used to treat infectious diseases come from herbs, such as *Honeysuckle*, *Isatis Root*, *Glycyrrhiza glabra*, and others. Moreover, there are numerous herbal products and extracts available, including *Scutellaria baicalensis*, *Dendrobium*, *Andrographis paniculata*, *Pelargonium*, and more (Table 1).

**Table 1:** Reported TCMs used for the prevention and treatment of infectious diseases.

TCM	Active compounds	Biological activity	Efficacy	Products	Reference
*Honeysuckle*	Chlorogenic acid, flavonoids, polysaccharides	Antiviral, anti-inflammatory	Treatment of infections caused by various viruses (including hepatitis B virus, adenovirus, influenza A virus, dengue virus, enterovirus, and respiratory syncytial virus); antimicrobial (*Staphylococcus aureus, Streptococcus haemolyticus, Escherichia coli, Bacillus dysenteriae, B. typhi, B. paratyphi, Pseudomonas aeruginosa, Klebsiella pneumoniae*, and *B. tuberculosis*); inhibition of pro-inflammatory cytokines, prevention of cytokine storms	Yin Qiao Jie Du Pian; Fu Fang Jin Yin Hua Ke Li	Gamaleldin Elsadig Karar et al. (2016), Tamura et al. (2006), Wang et al. (2009), and Elebeedy et al. (2021)
*Scutellaria baicalensis*	Baicalin, baicalein	Anti-inflammatory, antioxidant, anti-bacterial, anti-viral, immunomodulatory	Broad-spectrum antiviral activity against HIV, influenza disease, DENV, HBV and HTLV-I.	Huang Qin Jiao Nang	Jang et al. (2023), Li K. et al. (2021), Zandi et al. (2012), and Yang et al. (2014)
*Dendrobium*	polysaccharide	Antioxidant, immunostimulant	Antiviral activity against influenza A virus	/	Duan et al. (2022) and Li R. et al. (2017)
*Isatidis Radix*	Alkaloids, organic acids, flavonoids, volatile oils and polysaccharides	Antiviral	Inhibits the replication of human influenza viruses (H1N1 and H3N2), avian influenza viruses (H6N2 and H9N2), respiratory syncytial virus (RSV), and human herpes simplex virus type I (HSV-1), and shows antiviral activity against herpes simplex virus type II (HSV-2) and influenza A virus (IAV)	Ban Lan Gen Ke Li	Tong et al. (2020), Roy et al. (2018), Xiao et al. (2016), Li Z. et al. (2017), and He et al. (2014)
*Andrographis paniculata*	Diterpenoids, lactones and flavonoids	Anti-inflammatory, anti-bacterial, anti-viral, immunomodulatory	Antiviral (Influenza A virus (IAV), Human Immunodeficiency Virus (HIV), Hepatitis B and C, Herpes Simplex Virus I, EBV, etc.), Antibacterial (*S. aureus, P. aeruginosa, Shigella* spp., *Salmonella* spp., *Candida* spp.*, S. pneumoniae*, etc.)	Andrographolide Tablets, Andrographolide Drops, Andrographolide Injection	Jiang et al. (2021), Intharuksa et al. (2022), and Ding et al. (2022)
*Pelargonium*	Tannins, flavonoids, essential oil flavonoids and carboxylic acid compounds	Anti-inflammatory, anti-bacterial, anti-viral, anti-oxidant, immunomodulatory	Inhibits viruses (influenza virus, HIV, etc.), bacteria (*S. aureus, S. pneumoniae, S. enterica, P. aeruginosa*, etc.)	Geranium Essential Oil	Kong et al. (2023)
*Glycyrrhiza glabra*	Glycyrrhizin, glycyrrhizic acid, glycyrrhizinone A, glycyrrhizinone E	Antiviral, anti-inflammatory, antioxidant	Antiviral (HCV, HIV, CVB71, DHV, EV16, CVA5, HSV and H1N1, etc.), Antibacterial (*Mycobacterium tuberculosis*)	Compound Liquorice Tablets, Diammonium Glycyrrhizinate Capsules, Jin Sang Zi Hou Pian	Guan et al. (2020), Wang et al. (2015), and Derosa et al. (2021)
*Forsythia* koreana	Forsythoside, Forsythin, Isoforsythoside	Anti-inflammatory, antioxidant, antibacterial, antiviral	Antiviral (against influenza A virus and respiratory syncytial virus, etc.) Antibacterial (*E. coli, Bacillus, M. pneumoniae, B. dysenteriae, P. aeruginosa and S. aureus*, etc.)	Shuanghuanglian Injection, WeiCYinQiaoPian, Antiviral oral solution, etc.	Wang et al. (2018), Yuan H. et al. (2017), and Wu et al. (2016)
*Coptis chinensis*	Isoquinoline alkaloids	Antibacterial, antiviral, anti-inflammatory	Antibacterial (*S. aureus, S. pyogenes, B. anthracis, A. pleuropneumoniae, S. dysenteriae* and *E. coli*, etc.), cytokine storm sedation	Huang Lian Shang Qing Pian, Shuanghuanglian Oral Liquid	Yu et al. (2022), Batlle and Massagué (2019), and Pool (2003)
*Artemisia argyi*	Flavonoid glycosides, volatile oils, steroids, chromones, coumarins, phenolic acids, terpenoids	Antibacterial, anti-inflammatory, antioxidant, immunomodulatory	Antibacterial against *E. coli, S. enteritidis, P. aeruginosa, K. pneumoniae and S. aureus, C. albicans*	*Artemisia absinthium* volatile oil	Ding et al. (2021), Hurwitz et al. (2008), and Liu et al. (2021)
*Lilium longiflorum*	Steroidal saponins, sterols, polysaccharides, alkaloids and flavonoids	Anti-inflammatory, antioxidant, antimicrobial, immunomodulatory	Antioxidant damage	/	Sun et al. (2022) and Cai et al. (2015)
*Crocus sativus*	Saffronin and saffron aldehyde	Anti-inflammatory, antioxidant	Antioxidant damage	/	Gong et al. (2021)
*Cryptotaenia japonica*	Saikosaponin, steroidal saponins, flavonoids, coumarins, fatty acids, essential oils, polyacetylene and polysaccharides	anti-inflammatory	Suppressing damage from inflammation	Xiaochaihu granule	Jiang et al. (2020) and Xiang et al. (2020)

In China, *Honeysuckle* is widely utilized to treat viral upper respiratory tract infections caused by influenza, parainfluenza, respiratory syncytial virus, and others. It has demonstrated antiviral effects against HIV (Tamura et al., 2006; Gamaleldin Elsadig Karar et al., 2016), adenovirus, hepatitis B virus, herpes simplex virus, and more (Wang et al., 2009). Additionally, it possesses significant antimicrobial activity against strains of *Staphylococcus aureus*, *Streptococcus hemolyticus*, *Escherichia coli*, *Bacillus dysenteriae*, *B. typhi*, *B. paratyphi*, *Pseudomonas aeruginosa*, *Klebsiella pneumoniae*, and *B. tuberculosis* (Shang et al., 2011).

*Scutellaria baicalensis* is effective in the treatment of hepatitis, pneumonia, respiratory infections, and allergic diseases (Jang et al., 2023). Dried *S. baicalensis* exhibits antiviral properties and displays antiviral activity against HIV (Li K. et al., 2021), influenza virus, and HBV (Zandi et al., 2012).

*Dendrobium* contains alkaloids, polysaccharides, and terpenoids as its main components. Alkaloids are natural substances with significant biological activity, which are essential for the medicinal properties or toxicity of plants (Duan et al., 2022). Dendrobine, a constituent of *Dendrobium*, shows antiviral activity against influenza A virus by hindering the early stages of the viral replication cycle (Li R. et al., 2017).

*Isatidis Radix* is commonly used clinically for the treatment of influenza, colds, fever, hepatitis, encephalitis (Roy et al., 2018; Tong et al., 2020), and for preventing Severe Acute Respiratory Syndrome (SARS) in 2003 (Xiao et al., 2016). It specifically inhibits human influenza viruses (H1N1 and H3N2), avian influenza viruses (H6N2 and H9N2), respiratory syncytial viruses (RSV), and human herpes simplex virus type 1 (HSV-1) replication (Li Z. et al., 2017). *Isatidis Radix* has also exhibited antiviral activity against HSV-2 and influenza A virus (IAV), and is used for detoxification due to its significant antiviral effects (He et al., 2014).

*Andrographis paniculata* is commonly used for treating colds, fever, sore throat, cough, and respiratory symptoms (Jiang et al., 2021). It possesses the ability to eradicate various strains of viruses, including Influenza A Virus (IAV), Human Immunodeficiency Virus (HIV), Hepatitis B and C, Herpes simplex virus type I, and EBV (Intharuksa et al., 2022). *Andrographis paniculata* lactone is clinically employed to treat bacterial infections and stimulates an immune response that aids in combating microbial infections by regulating its complementary system, granulocytes, and macrophages (Mishra et al., 2021; Zhang H. et al., 2021).

The polyphenolic methanolic extract of *Pelargonium* exhibits antiviral activity against influenza virus and reduces the infectivity of different types of influenza viruses in laboratory settings. *Pelargonium* also demonstrates a wide range of antimicrobial effects, including inhibitory effects against *S. aureus*, *E. coli*, *Salmonella enteritidis*, and more (Kong et al., 2023).

*Glycyrrhiza glabra* displays broad-spectrum antiviral and immunomodulatory effects against various strains of influenza viruses (Guan et al., 2020). It possesses antiviral activity against HCV, HIV, CVB71, DHV, EV16, CVA5, HSV, and H1N1 (Wang et al., 2015).

*Forsythia koreana* is an antimicrobial agent with a broad range of activity primarily used in the treatment of upper respiratory tract infections and acute nephritis. It can hinder the growth of *E. coli*, *Mycoplasma pneumoniae*, *B. dysenteriae*, *P. aeruginosa*, and *S. aureus* (Wang et al., 2018).

*Artemisia argyi* exhibits inhibitory activity against pathogens like *E. coli*, *S. enteritidis*, *P. aeruginosa*, *K. pneumoniae*, *S. aureus*, *Candida albicans*, and *Aspergillus niger*. This activity is attributed to the high levels of 1, 8-naringeno and β-siderone present in *A. argyi*. The entire plant of *A. argyi* and its leaf extracts have also shown antimicrobial properties (Ekiert et al., 2020).

### 2.2. Compounds in Chinese herbal medicines that exert medicinal effects against infectious diseases

Chinese herbal medicines contain various active ingredients such as alkaloids, flavonoids, polysaccharides, saponins, tannins, and polyphenols. Common alkaloid-containing components in Chinese medicines include picloram, ephedrine, and berberine. Flavonoid-containing components include baicalein, puerarin, and total flavonol glycosides. Saponin-containing components include licorice glycosides, chaihu saponins, and ginsenosides. These active ingredients enable Chinese medicines to effectively treat infectious diseases by exerting antiviral, antimicrobial, anti-inflammatory, immunomodulatory, and antioxidant effects.

For instance, *Honeysuckle* contains active substances such as chlorogenic acid, dicaffeoylquinic acid, and bisabolol (Shang et al., 2011; Jang et al., 2023). *Scutellaria baicalensis* mainly consists of baicalin and baicalein. *Dendrobium* contains alkaloids, polysaccharides, and terpenoids. *Andrographis paniculata* contains diterpenoids, lactones, and flavonoids. *A. argyi* contains flavonoid glycosides, volatile oils, steroids, chromones, coumarins, phenolic acids, terpenoids, and other constituents (Ding et al., 2021). *Glycyrrhiza glabra* includes licorice sweeteners, 18β-glycyrrhizinic acid, glycyrrhizin, glycyrrhizin aldosterone A, glycyrrhizin aldosterone E, and photoglycyrrhizin.

### 2.3. Pharmacodynamics and pharmacokinetics of herbal medicines against infectious diseases

Pharmacokinetics refers to how a drug moves through the body, including absorption, distribution, metabolism, and excretion. The specific responses to a drug depends on its pharmacologic properties at its site of action. Resveratrol, found in many medicinal herbs, has a unique pharmacokinetic profile. It has shown inhibitory activity against viral replication and virus-induced inflammation in diseases caused by various pathogenic human viruses, such as influenza viruses, human coronaviruses, respiratory syncytial viruses, rhinoviruses, and human metapneumovirus.

However, resveratrol is rapidly and extensively metabolized, resulting in low bioavailability in its unmetabolized form in the plasma. It is poorly soluble above pH 7.4. When taken orally, about 77–80% is absorbed in the gastrointestinal tract, and around 49–60% of the unabsorbed portion is excreted in the urine. In the intestine and liver, resveratrol undergoes rapid metabolism to produce metabolites with low bioactivity, including sulfates and glucosinolate affixes.

Recent studies have also highlighted the significant role of gut microbiota in resveratrol metabolism, influencing its bioavailability. Gut bacteria can promote resveratrol synthesis from its precursors or affixes, as well as metabolize resveratrol to dihydroresveratrol, which can be absorbed, bound, and excreted.

In the bloodstream, resveratrol can exist as glucosinolates, sulfates, or unmetabolized form. However, the limited bioavailability of unmetabolized resveratrol is due to its binding to albumin and lipoproteins, forming complexes that act as polyphenol reservoirs. In the human body, an initial dose of 25 mg results in plasma concentrations ranging from 1 to 5 ng/mL, while higher doses (up to 5 g) can lead to resveratrol plasma concentrations as high as 530 ng/mL. Due to the rapid metabolism and poor bioavailability of resveratrol, researchers are exploring ways to enhance its therapeutic efficacy, such as administering very high oral doses (Filardo et al., 2020).

## 3. Mechanisms of Chinese herbs against infectious diseases

### 3.1. Antiviral mechanism

#### 3.1.1. Inhibition of viral replication

**Figure 2:**
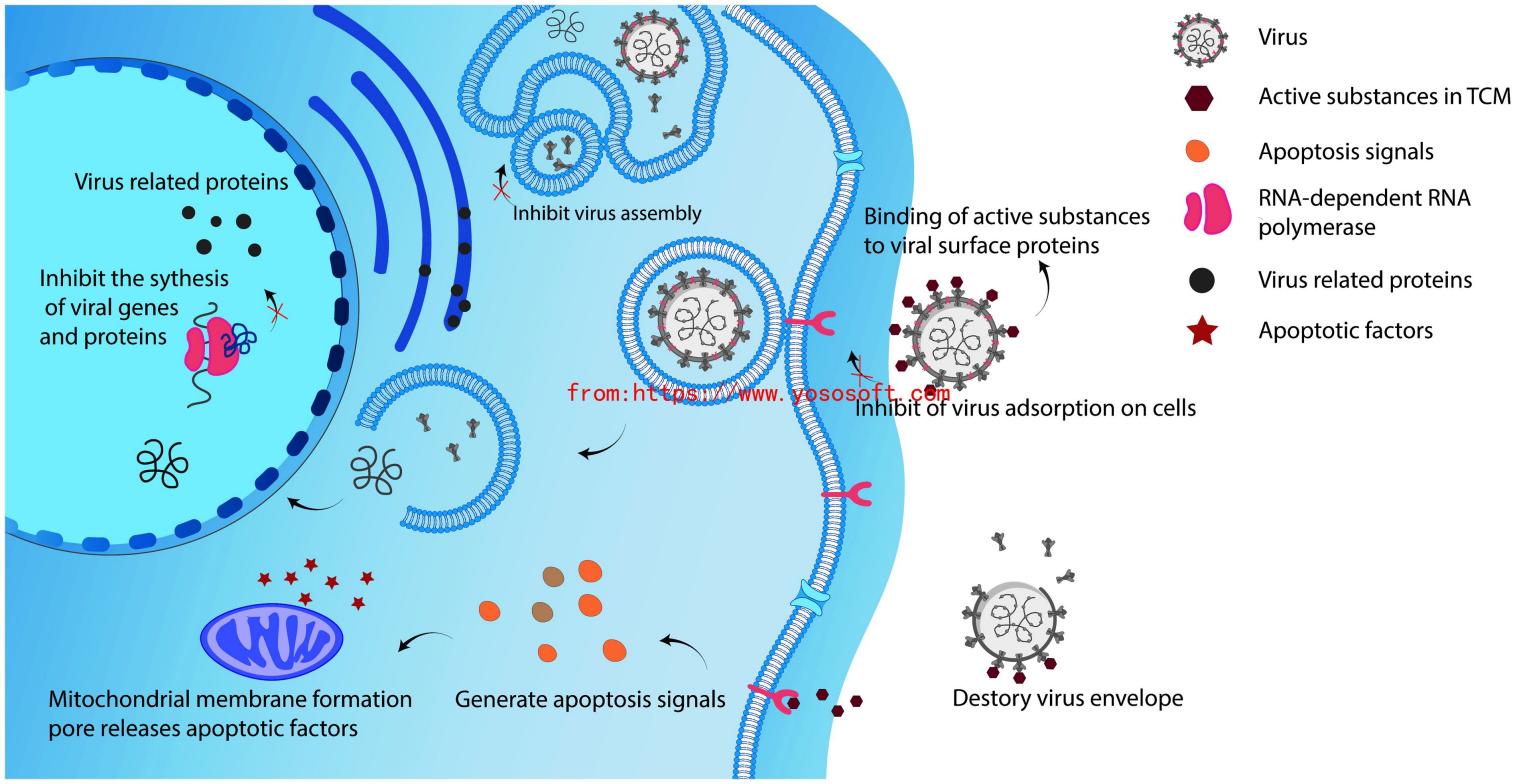
TCM inhibits the process of virus replication.

Chinese herbs have been discovered to hinder the replication of viruses through various mechanisms, primarily related to three stages: interfering with viral invasion, hindering gene replication, and obstructing the synthesis of fully functional proteins (Figure 2; Jiang et al., 2021; Chen and Ye, 2022). Extracted active components from these herbs, such as alkaloids, flavonoids, saponins, quinones, terpenes, proanthocyanidins, tannins, polysaccharides, steroids, polyphenols, and coumarins, have shown the ability to scavenge, inhibit DNA and RNA synthesis, and impede viral replication (Abookleesh et al., 2022). The antiviral effects of these components are achieved by inhibiting these three phases.

Viral attachment and entry are therapeutic targets for herbal medicines in the viral life cycle. The SARS-CoV-2 virus uses the receptor-binding domain of its glycosylated S protein to bind to human angiotensin-converting enzyme 2 (ACE2) and initiate membrane fusion and viral entry. Tanshinone IIA and tanshinic acid B, which are compounds found in the TCM *Salvia miltiorrhiza*, have been found to inhibit viral entry into cells through interactions with specific drug targets (Elebeedy et al., 2021). During the viral replication phase, the main drug targets of SARS-CoV-2 include 3-cancreatine lactamase-like protease (3CLpro), papain-like protease (PLpro), RNA-dependent RNA polymerase, and spike protein. While quercetin interferes with the replication ability of SARS-CoV-2 by inhibiting 3CLpro and PLpro (Derosa et al., 2021).

Among these components, alkaloids are the largest group of antiviral compounds, possessing a wide range of antiviral properties. Flavonoids present in honeysuckle extract inhibit the activity of neuraminidase in influenza viruses. Neuraminidase plays a vital role in releasing viruses from host cells, so inhibiting its activity prevents the replication and release of influenza viruses once they enter host cells (Li M. et al., 2021). Glycyrrhizin sweetener and glycyrrhizinic acid bind to the surface of host cells through ACE 2. The spike protein is then cleaved into the S1/S2 domain by the host transmembrane serine protease 2 (TMPRSS2). The S1 domain contains the receptor-binding domain (RBD) that directly binds to ACE 2. After cleavage of the spike protein, the virus enters the cell through fusion of the virus and cell membrane, exhibiting an antiviral effect (Diomede et al., 2021).

Moreover, herbal medicines not only inhibit virus proliferation within cells, but also affect the chemical composition of the virus by modifying the structure of viral surface proteins. This reduces or even eliminates the virus’s capability to attach to cellular receptors and infect cells. In conclusion, Chinese herbs hinder viral replication by interrupting the process from viral entry into the cell to self-replication, thus achieving the effect of inhibiting viral replication.

#### 3.1.2. Induction of apoptosis

Chinese herbal medicines have a significant role in treating infectious diseases through the promotion of apoptosis in virus-infected cells, which helps limit viral replication and spread (Turpin et al., 2022). Additionally, these medicines can activate immune responses and assist in the production of antiviral immune responses. Chinese herbal medicines contain natural plant compounds like flavonoids and alkaloids, which possess apoptosis-inducing activities either directly or indirectly. This is because many molecules involved in the apoptotic pathway in cells are capable of regulating various cellular behaviors, including cell growth and apoptosis. An experimental proof was conducted using vascular smooth muscle cells that were stimulated by interleukin-1 as a model (Rocchetti et al., 2023). The use of fluorescence microscopy allowed for the observation of apoptotic body formation and chromosome aggregation. It was confirmed that puerarin and icarosin, both found in flavonoids, can promote VSMC apoptosis at low concentrations (10^–7^ mol/L). Additionally, the quantification and comparison of T cell apoptosis and mRNA expression levels were carried out using TUNEL labeled flow cytometry and fluorescence real-time quantitative PCR technology. The results indicated that total flavonoids have a role in regulating T cell apoptosis and related genes in corticosterone rats (Liu et al., 2020). The active components found in Chinese herbs induce apoptosis by regulating apoptotic protein families such as the Bcl-2 family and the Caspase family, as well as impacting apoptosis signaling pathways like the p53 pathway and the NF-κB pathway (Yu et al., 2022).

For instance, baicalein, a flavonoid compound present in *S. baicalensis Georgi*., can induce apoptosis by suppressing the expression of E6 and E7 viral oncogenes in cervical cancer cells infected with human papillomavirus (HPV), such as CaSki and SiHa cells (Kim et al., 2013). Self-apoptosis in infected cells can block the spread of intracellular viruses.

### 3.2. Antimicrobial mechanism

#### 3.2.1. Inhibition of bacterial growth

The ability of Chinese herbs to inhibit bacterial growth lies in their interference with bacterial metabolic processes. Substances like flavonoids and flavonoid glycosides found in these herbs can hinder enzyme activity in bacteria and disrupt important biological processes such as protein and nucleic acid synthesis, thereby impeding bacterial metabolic pathways and preventing their growth. Flavonoids can also impede nucleic acid synthesis, cytoplasmic membrane function, and energy metabolism (Biharee et al., 2020). Furthermore, specific herbs contain active compounds that directly damage bacterial cell walls, membranes, and internal components, leading to the deactivation and death of bacteria. This direct interference with bacterial survival mechanisms further inhibits bacterial growth. One particular compound, chlorogenic acid, can disrupt the normal progression of the cell cycle and impact cell growth (Majumder et al., 2020).

The antimicrobial effects of these herbs are attributed to various mechanisms, including cell membrane disruption, inhibition of protein and DNA synthesis, inhibition of bacterial division and development, and inhibition of cytokinin (Wang et al., 2019).

#### 3.2.2. Disruption of bacterial biofilm

Certain herbs contain active ingredients like xanthophylline, xanthophyllin, and flavonoids that can effectively dissolve bacterial biofilm. This action disrupts the biofilm’s structure, preventing bacteria from forming a healthy biofilm and impacting their attachment and survival (Wang et al., 2019). Moreover, these active ingredients can also interfere with the bacterial signaling system and gene regulatory network, inhibiting biofilm formation. Bacterial biofilms mostly consist of polysaccharides, lipids, and proteins (Flemming and Wingender, 2010; Strahl and Errington, 2017). Biofilm formation is closely linked to bacterial growth and survival, as the matrix surrounding bacteria helps them resist antimicrobial treatments. Flavonoids, for example, play a vital role in inhibiting bacterial growth by reducing adhesion, biofilm formation, pore proteins on cell membranes, membrane permeability, and pathogenicity. The generation of energy within the bacteria is inhibited, the activity of intrabacterial enzymes is inhibited, and bacterial nucleic acid synthesis is inhibited (Gupta et al., 2022). Emodin reduces the growth of *S. aureus* biofilm. Its antimicrobial mechanism against *S. aureus* is associated with intervening in the release of extracellular DNA and down-regulating the expression of biofilm-associated genes, including cidA, icaA, dltB, agrA, sortaseA, and sarA (Zheng et al., 2021). In the case of *Pseudomonas aeruginosa*, andrographolide has been found to completely inhibit biofilm formation (Jiang et al., 2009). The antibacterial effects of chlorogenic acid are achieved by disrupting the synthesis of bacterial cell membranes, resulting in the loss of cellular contents and bacterial inactivation. It also disrupts the structure of the outer membrane, inner membrane, and cell wall of *S. enteritidis*, causing leakage of cellular contents and exhibiting antibacterial effects against *Salmonella enteritidis* (Majumder et al., 2020). By disrupting bacterial biofilm, the pathogenicity and growth of bacteria are reduced, making the lysing of bacterial biofilm a mechanism of antimicrobial activity in herbs.

### 3.3. Immunomodulatory mechanisms

#### 3.3.1. Regulation of immune cell activity

**Figure 3:**
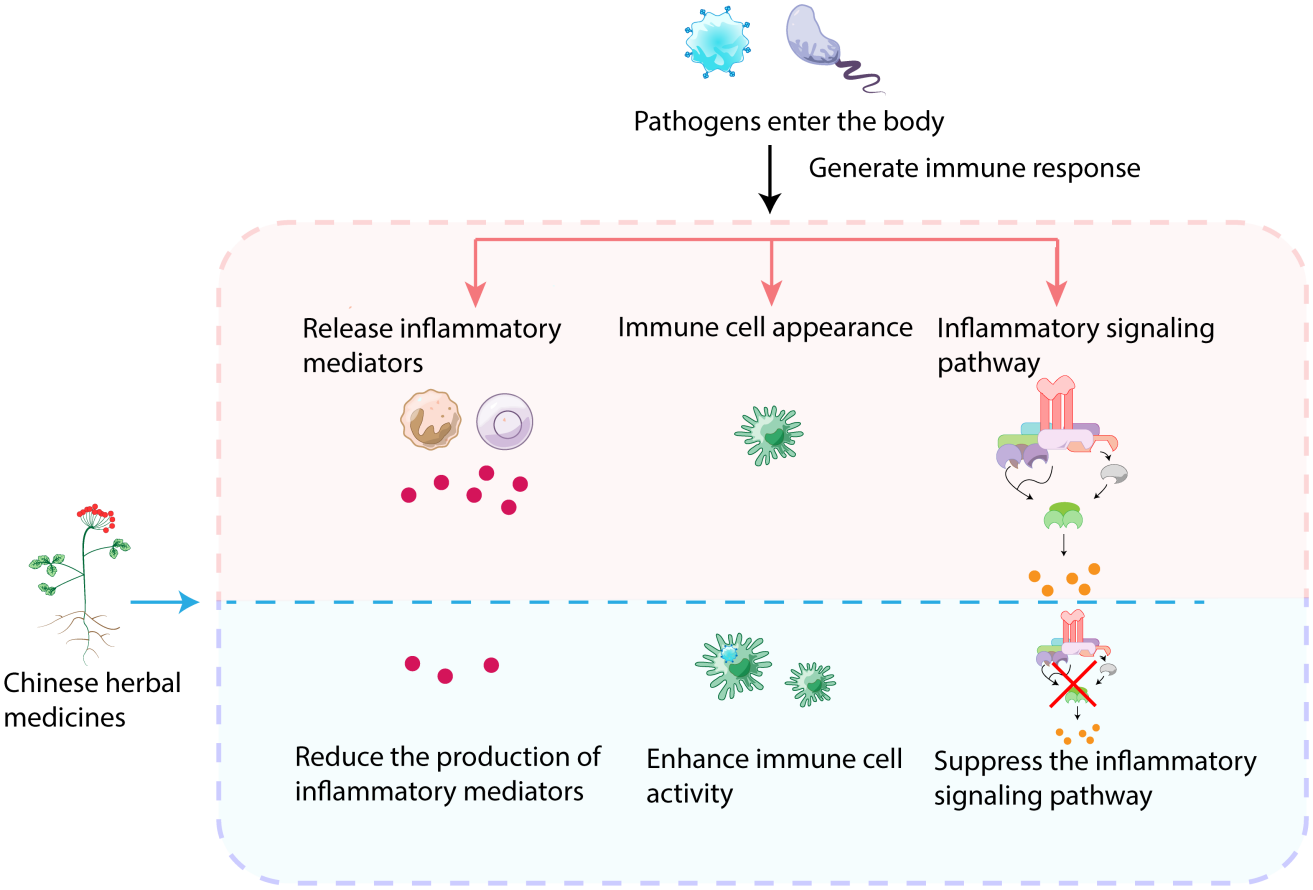
Immunomodulatory mechanism of TCM.

Herbal medicines have the ability to regulate the immune response by balancing the over-activation or suppression of the immune system (Figure 3). This helps in reducing tissue damage and inflammatory responses during bacterial infections. Herbs can modulate immune cell activity and enhance the function of macrophages, natural killer cells, and lymphocytes (Yang et al., 2021), making them more effective in eliminating pathogens and enhancing immune cell activity. Additionally, they inhibit the growth and reproduction of pathogens, promote synergistic interactions between immune cells, and regulate the production and release of immune factors like interferons, cytokines, and inflammation-modulating factors (Zhang Q. H. et al., 2021). Components such as polysaccharides, flavonoids, saponins, and phenolic compounds present in Chinese herbs can regulate immune-related molecules to exert immunomodulatory effects during the fight against microorganisms such as viruses and bacteria. These active ingredients also inhibit the overactivation of macrophages and neutrophils (Li et al., 2015). For instance, polysaccharides regulate inflammatory cytokines such as IL-1β, IL-6, TNF-α, and IFN-γ, as well as the expression of inducible nitric oxide synthase, thereby increasing antiviral activity and preventing macrophage overactivation (Ohta et al., 2007). Polysaccharides have the ability to bind to pattern recognition receptors important in innate immunity, thus modifying immunity through binding (Lee et al., 2020). Honeysuckle polysaccharides, for example, can modulate nonspecific immunity. Saponin-containing adjuvants have been found to alter lymphatic flow and facilitate antigenic entry into draining lymph nodes in a mast cell-dependent manner. The immunoreactivity of saponins is influenced by specific functional groups (Silva et al., 2021). Herbs can regulate the activity of immune cells in a two-sided manner; they promote immune cell activity when the body is infected, but they exhibit an inhibitory effect when the cells are overactivated.

#### 3.3.2. Inhibition of immune-related mediator release

Herbal medicines are capable of modulating inflammatory modulators as they can inhibit the release of inflammatory mediators, cytokines, and other factors that are generated during the inflammatory response. This ultimately decreases inflammation (Chen et al., 2018). Inflammation is a complex biological response to harmful stimuli that protects organisms from pathogens. However, if inflammation becomes excessive or uncontrolled, it can result in harmful tissue damages and trigger the release of inflammatory mediators.

For instance, individuals with COVID-19 often have notably elevated levels of cytokines, including IL-2, IL-6, IL-7, G-CSF, CXCL10, CCL2, CCL3, and TNF-α (Huang et al., 2020). Artemisinin, derived from *A. annua L.*, has the ability to impact various points within the immune signaling cascade specifically targeting activated pathogenic T cells. This leads to a synergistic therapeutic effect on disease activity (Shi et al., 2015), and it also reduces systemic levels of inflammatory cytokines during infection (Uckun et al., 2021). Chaihu saponin could potentially possess anti-inflammatory effects by directly inhibiting the expression of pro-inflammatory cytokines and modulating inflammatory mediators. *Salvia miltiorrhiza* can decrease the inflammatory response by lowering the levels of inflammatory cytokines IL-6β, IL-55, and TNF-α. Forsythiaside, found in Forsythiae fructus, decreases the release of IL-6, IL-1β, TNF-α, and COX-2 by down-regulating the mRNA expression of nuclear NF-κB. The isoflavonoids genistein, stomatophorol, bitter acacia glycosides, and genistein isolated from *Fructus sophorae* have demonstrated significant activity against the primary pro-inflammatory mediator interleukin IL-5 (He et al., 2016). *Scutellaria baicalensis* can inhibit the production of inflammatory factors and reduce the levels of TNF-α, IL-6, chemokines, and other inflammatory mediators, thereby decreasing the extent of the inflammatory response (Liao et al., 2021). Through the inhibition of immune-related factor release, herbs have the ability to lessen the damage inflicted on the body when there is an excess of inflammatory mediators.

#### 3.3.3. Inhibition of immune-related signaling pathways

Active ingredients found in Chinese herbs can regulate immune-related signaling pathways, effectively intervening in the activation and transduction processes of inflammatory signaling pathways, and inhibiting the activity of inflammation-related signaling pathways like NF-κB and AP-1 (Batlle and Massagué, 2019). As a result, this reduces inflammatory responses (Borges et al., 2019).

Flavonoids, for instance, have the ability to modulate various intracellular signaling pathways, such as NF-kB mitogen-activated protein kinase, by interfering with the redox-sensitive network of immune cells. Baicalin inhibited NF-κB by means of suppressing its phosphorylation and nuclear translocation of p65, and regulated the upstream of TLR4 in two approaches: it suppressed the expression of HMGB1, or it bound to the hydrophobic region of MD-2 and inhibited the formation of the MD-2/TLR4 complex to inhibits the TLR4/NF-κB pathway to suppress highly activated inflammatory responses (Jiang et al., 2020). *Andrographis paniculata* may inhibit inflammation through the inhibition of the NF-κB/MAPK signaling pathway (Li Y. et al., 2017). Puerarin is involved in inhibiting inflammatory responses by reducing the levels of TNF-α and IL-1β through the inhibition of NF-κB signaling induced by puerarin (Yuan et al., 2016). Picrasidine exerts anti-inflammatory effects by activating or inhibiting specific key molecules in multiple cell signaling pathways (Lin et al., 2022). The anti-inflammatory effects of *F. fructus* are associated with the inhibition of NF-κB, MAPK, and JAK-STATs signaling pathways, along with the activation of Nrf2 signaling (Wang et al., 2018). The molecular mechanisms behind the anti-inflammatory effects of the isoquinoline alkaloids found in *Rhizoma coptidis* include the down-regulation of Toll-like receptors (TLRs) and inflammation-associated pathways. These pathways include nuclear factor-κB (NF-κB), mitogen-activated protein kinase (MAPK), Janus kinase/signal transducer and activator of transcription (JAK/STAT), and inflammatory vesicle NLRP3 (Lan et al., 2022). The flavonoid isoglycyrrhizin in *G. glabra*, used to treat excessive and persistent inflammation caused by *Mycobacterium tuberculosis* infection, induces inhibition of Notch1/NF-κB and MAPK signaling pathways. This also presents new avenues for exploring the anti-inflammatory mechanisms of ISL (Sun et al., 2022). *Radix Bupleuri* exhibits anti-inflammatory activity by modulating inflammation-related signaling pathways, such as the nuclear factor-κB (NF-κB) pathway and mitogen-activated protein kinase (MAPK) pathway (Yuan B. et al., 2017). Through the regulation of immune signaling pathways, herbs are capable of transcribing several cytokines to mediate an immune response in order to eliminate invading pathogenic bacteria.

### 3.4. Antioxidant effects

Microbial infections lead to infectious diseases, which in turn result in oxidative stress. Numerous herbs contain antioxidant substances that can scavenge free radicals and reduce oxidative stress, thus lessening inflammatory responses and tissue damages (Cui et al., 2018). For instance, saffron has anti-inflammatory effects by modulating the oxidative stress defense system and scavenging free radicals (Bastani et al., 2022). *Forsythia koreana* contains lignans that protect HDL and LDL from lipid peroxidation, and *in vitro* studies have demonstrated the ability of forsythia glycosides, calyculol glycosides, and ethanolic extracts of Forsythia to scavenge free radicals (Wang et al., 2018). Many active ingredients found in traditional Chinese medicine, such as natural polysaccharides, polyphenols, and flavonoids, possess antioxidant activity. Natural polysaccharides exert antioxidant effects by modulating signal transduction pathways, regulating enzyme activities, or scavenging free radicals or substances that are prone to free radical formation (Chen et al., 2021; Bai et al., 2022). Polysaccharides present in lilies can act as electron or hydrogen donors to eliminate harmful hydroxyl radicals (Gao et al., 2022). Phenolics resist oxidative damage by eliminating excess activated oxygen or directly removing free radicals and the enzymes that produce them (Li D. et al., 2021). Flavonoids reduce reactive substances by inhibiting enzymes involved in oxidative mechanisms or by regulating ion channels (Al-Khayri et al., 2022). *Crocus sativus* also demonstrates anti-inflammatory effects by modulating the oxidative stress defense system to scavenge free radicals (Bastani et al., 2022). The active substances present in herbs are capable of reducing oxidative reactions caused by infections in the body through the removal of excess free radicals and the activation of oxygen.

### 3.5. Synergistic effects and potential mechanisms of TCM in the prevention and control of infectious diseases

The combination of two or more drugs to produce a greater overall effect than the sum of their individual effects is known as synergism (Zhou et al., 2016). The theoretical basis of synergism is based on two main aspects. Firstly, it can enhance the effectiveness of herbal extracts by improving their bioavailability and reducing adverse effects through multi-target effects (Yang et al., 2014). When treating infectious diseases, combining herbs can enhance their efficacy (Ding et al., 2022). This synergistic effect may be attributed to the formation of new compounds during the preparation of the herbs (Yuan H. et al., 2017). For instance, the Sanhuang laxative heart soup, made from *rheum officinale*, *Coptis chinensis*, and *S. baicalensis*, is a well-known formula used for combating inflammation. The main components of this formula, such as rhubarbine, berberine, and baicalin, can improve the absorption or decrease the removal of other components, ultimately synergistically influencing the inflammatory process and enhancing the formula’s anti-inflammatory effects (Wu et al., 2016).

Complementary medicinal effects: multiple herbs with different compositions and mechanisms of action can complement each other to achieve a more comprehensive therapeutic effect. For example, one herb may inhibit the production of inflammatory factors, while another can scavenge free radicals. The combination of these two herbs can simultaneously inhibit the inflammatory responses and attenuate oxidative damages. A well-known example is the simultaneous use of multiple drugs for synergistic treatment of HIV infection (Pool, 2003; Hurwitz et al., 2008).

## 4. Challenges

**Figure 4:**
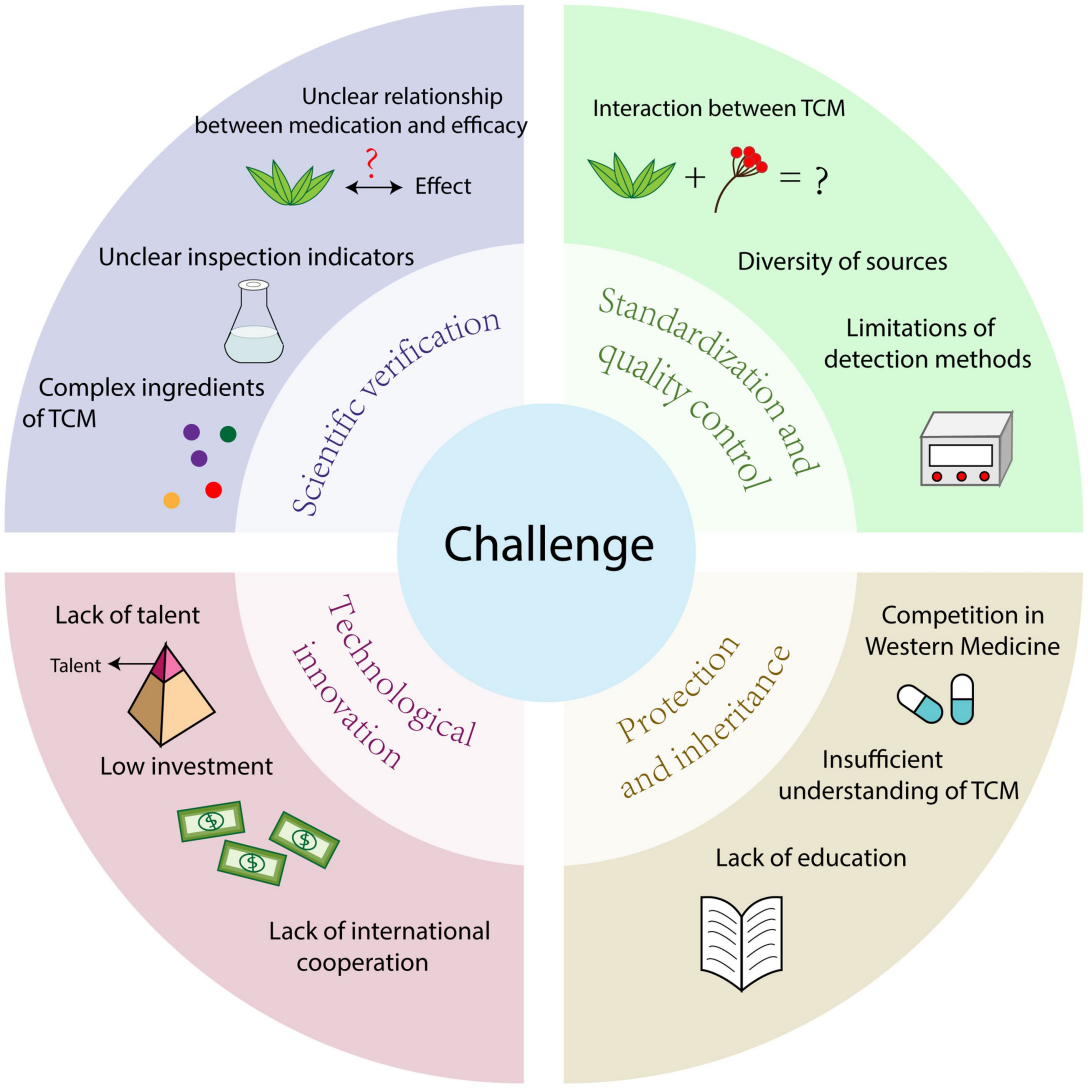
Challenges faced by TCM in the fight against infectious diseases.

TCM has good effect on the treatment of infectious diseases, how to give full play to the advantages of TCM in disease prevention and treatment has become a major problem in front of researchers, and the development of TCM is still facing many challenges (Figure 4).

### 4.1. Lack of scientific validation

Most Chinese medicines are based on the experiences and knowledge of TCM practitioners and scholars, often lacking scientific validation. The effectiveness of TCM relies on the complex combination of multiple components and the principle of holistic treatment, which differs from the mechanism of action of Western medicines that often consist of single components. Throughout China’s history, TCM has demonstrated its efficacy through extensive clinical practice. However, the theories behind TCM may be difficult to explain for individuals with orthodox medical training (Liu et al., 2021). This is where clear scientific validation and support from clinical data become crucial.

The diverse chemical composition of TCM and the complex interactions between herbs and the human body result in limited information on the pharmacokinetics, pharmacodynamics, effectiveness, and safety of TCM. The lack of scientific evidence regarding therapeutic efficacy is a major concern for health authorities and the public (Cai et al., 2015). The complexity of TCM makes it challenging to accurately assess their therapeutic effects and mechanisms in research. Scientific validation requires significant investment of time, money, and resources, but currently, minimal resources are being allocated to this area.

Many of the chemical substances present in TCM lack appropriate test indicators, leading to test results that do not fully and comprehensively reflect the quality of TCM. When China established indicators for examining the quality of TCM, the correlation between these indicator substances and the effectiveness of the medicines was not clarified. Furthermore, understanding the relationship between drug administration and therapeutic efficacy remains a significant challenge (Gong et al., 2021). With the growing emphasis on disease prevention and treatment, safety issues with TCM have become a bottleneck hindering the modernization of Chinese medicine.

### 4.2. Standardization and quality control

The quality of TCM is a significant concern because it directly affects the safety and effectiveness of its clinical use. In particular, the safety of herbal materials is crucial in clinical applications (Xiang et al., 2020), as there is a potential for adverse reactions that can jeopardize the medication’s safety (Xiang et al., 2020). Furthermore, these issues also lead to problems such as the inaccurate identification of herbal materials.

In addition to safety concerns, the current regulatory oversight may not meet the expectations for the quality and efficacy of herbal preparations (Wu et al., 2019). The lack of quality control has resulted in problems like the contamination of TCM. In some places, herbs are freely sold in markets without regulation, leading to mutual contamination and adverse effects for users (Chowell and Rothenberg, 2018). Therefore, quality control has become a significant issue in the modernization of TCM, with four main problems identified.

Firstly, the complexity of composition in TCM means that the interaction between different active substances can affect the drugs’ efficacy. This confounding medicinal effect may be related to changes in the composition of active substances (Zimmermann-Klemd et al., 2020). Additionally, different processing methods and effective parts of herbs can also influence the drugs’ efficacy. As some active substances in Chinese herbs are still unknown, establishing clear quality control indicators and standards is challenging (Hu et al., 2021).

Secondly, due to China’s vast land, the same type of TCM can vary depending on the natural conditions of the region, including differences in variety, quantity, and quality of TCM resources (Zhou et al., 2021). These geographic variations raise concerns about authenticity, quality, and efficacy, as well as market order issues and the risk of counterfeit TCM. Ensuring quality control of the composition and activity of these herbs has become a major focus.

Thirdly, the lack of uniform international standards for TCM hinders its widespread promotion and international development.

Finally, the limitations of testing methods may lead to inaccurate results when evaluating Chinese medicines. This, combined with the inadequacy of current quality control methods, is not compatible with the multi-target and multi-component characteristics of TCM and its production process (Liu et al., 2021). Lack of standardization and quality control creates distrust in the safety of TCM, impeding its overall development. Therefore, improving the quality standard system, promoting standardization, and enhancing the level of quality control is essential to advance TCM.

### 4.3. Insufficient cultural protection and inheritance

TCM is not only a medical science but also a cultural heritage that the Chinese people have honed and preserved for thousands of years. Although the use of Chinese herbs in preventing infectious diseases has immense potential, the current efforts to protect and pass on this knowledge have not met expectations. Inheritance is crucial for the development of Chinese medicine, but the divergence in concepts between Chinese medicine and modern medicine, along with the lack of public awareness and trust, puts the future of Chinese medicine at risk. Chinese medicine theories, prescriptions, and talents face significant challenges compared to Western medicine. However, safeguarding and promoting the development of Chinese medicine culture has become a pressing concern.

The protection and sustainable utilization of medicinal plants are essential for the advancement of the TCM industry. To support the innovation and preservation of Chinese medicine, a comprehensive set of policy measures is necessary. The lack of research data, control and regulatory mechanisms, expertise in national health authorities and regulatory bodies, information sharing, and adequate education and training of TCM practitioners all hinder the transmission of TCM (Su et al., 2021). Moreover, education serves as the foundation of TCM’s inheritance, and higher education in TCM disciplines plays a significant role. However, with the development of western medicine education, TCM education has also been affected. Therefore, traditional Chinese medicine education needs to gradually align itself with its own education law, emphasizing its unique characteristics and advantages. Currently, knowledge is one of the most crucial resources for social progress and economic development, yet the state’s protection of traditional Chinese medical knowledge remains insufficient (Lin et al., 2016). All these factors directly impact the inheritance of Chinese herbal medicine.

### 4.4. Lack of technological innovation

China’s current market performance in TCM is unsatisfactory, despite it being the birthplace of TCM. The lack of innovation power and scientific research talents in TCM result in China having only 0.3% of patents and intellectual property rights for TCM. Moreover, the form of TCM primarily being prescriptions makes it challenging to administer to certain groups such as infants, young children, patients with impaired consciousness, and comatose patients. Additionally, the process of decocting TCM is time-consuming and labor-intensive, which does not align with the fast-paced modern lifestyle and fails to fully meet the clinical needs for treating modern diseases. Therefore, technological innovation is crucial for improving TCM.

Furthermore, TCM and modern medicine research and development (R&D) lack sufficient integration. There is a shortage of important products that address new medical needs and utilize new technology. The pace of innovation in TCM is slower compared to Western medicine, resulting in inferior competitiveness. China invests less in scientific and technological innovation for Chinese herbal medicines compared to Western medicine. This limited funding, along with the need for enhanced R&D capabilities and facilities in research institutions and enterprises, hampers scientific research and technological innovation in Chinese herbal medicines.

International cooperation in TCM science and technology innovation is relatively limited in China. Encouraging international collaboration can facilitate cross-border exchanges and resource sharing, elevating the level and impact of scientific and technological innovation in Chinese herbal medicines.

## 5. Prospect and conclusion

**Figure 5:**
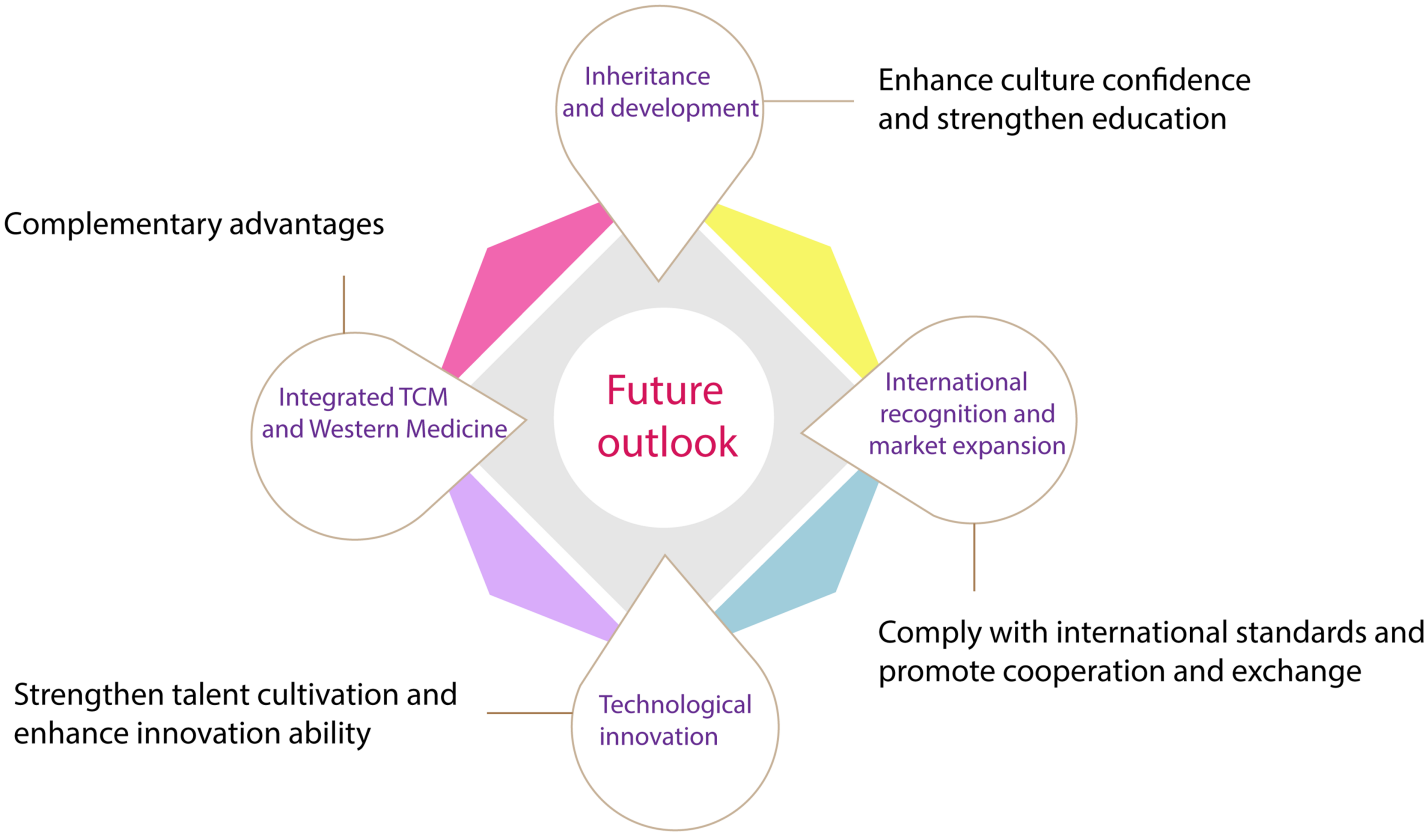
Prospects for the development of TCM on treating infectious diseases.

Due to its extensive activity and reliable safety, TCM has a long history of clinical application. The main active ingredients in TCM exhibit antiviral, antibacterial, and immune-regulating effects, making it highly promising in the field of infectious diseases. It is speculated that the combined use of TCM can result in a synergistic effect, enhancing its therapeutic effectiveness. With continuous research, it is believed that TCM will be able to address a wider range of medical issues in the future. This article aims to provide prospects for the challenges faced by TCM in terms of inheritance, innovation, internationalization, and integration of Chinese and Western medicine. The goal is to promote the development of TCM and its application in daily life (Figure 5).

### 5.1. Inheritance and development of Chinese medicine

The foundation for the inheritance and development of Chinese medicine is provided by TCM. These ancient Chinese science materials are treasures that have had a positive impact on the progress of world civilization. To ensure the continued growth of TCM, it is necessary to improve the service system and fully utilize the unique role of Chinese medicine in maintaining and promoting people’s health. Moreover, efforts should be made to strengthen the construction of Chinese medicine talents and focus on discovering and promoting young and middle-aged key talents and inheritors. To inherit and develop Chinese medicinal materials in an open and innovative way, it is necessary to strengthen the research and utilization of relevant classics, accelerate the scientific research and innovation of Chinese medicinal materials, and promote the open development of Chinese medicinal materials, so as to guarantee their future development.

### 5.2. Technological innovation and modernized production

Research on TCM will become more in-depth and systematic with advancements in science and technology. The promotion of herbal medicine bases and large-scale, standardized cultivation of herbal medicine is required to meet higher quality standards for TCM. The state will also prioritize strategic needs and major scientific problems in Chinese medicine by establishing multidisciplinary research platforms and accelerating the development of new Chinese medicine research, advanced Chinese medicine equipment, and Chinese medicine pharmaceutical equipment. These efforts will provide strong technical support for the development and protection of TCM.

### 5.3. International recognition and market expansion

Gaining international recognition involves evaluating the clinical advantages of TCM and addressing the lack of clinical evidence. To achieve this, TCM should be integrated into international cooperation programs such as the construction of the community of human destiny and the “Belt and Road” initiative. Special international cooperation programs for Chinese herbal medicine should be implemented, and international standards for TCM should be developed. Active participation in the development of international rules related to TCM and the promotion of TCM culture overseas are also necessary to expand the influence and competitive advantage of TCM on the international stage. Additionally, the service trade of TCM should be vigorously developed by encouraging the establishment of high-quality overseas centers, international cooperation bases, and service export bases, which will expand the market and foster the integrated development of TCM.

### 5.4. Combination of Chinese and Western medicine

The combination of Chinese and Western medicine plays a crucial role in ensuring the nation’s vitality. It is a strategic decision that brings together the advantages of both medical systems, complementing each other to provide better healthcare. This combination also presents an important opportunity for the development of Chinese medicine. Despite being rooted in ancient practices, Chinese medicine concepts are not outdated. By integrating modern Western medicine, Chinese medicine can adapt to the current healthcare landscape while preserving its heritage and fostering innovation. This approach benefits not only the Chinese people but also contributes to the global community.

Chinese medicine is currently flourishing and expanding in the best possible environment for its own development.

## Author contributions

QZ: Data curation, Formal analysis, Investigation, Methodology, Resources, Software, Validation, Writing – original draft, Writing – review & editing. YC: Data curation, Formal analysis, Investigation, Methodology, Software, Validation, Visualization, Writing – original draft. HQ: Data curation, Formal analysis, Investigation, Methodology, Software, Writing – original draft. RT: Data curation, Investigation, Software, Visualization, Writing – original draft. TH: Data curation, Formal analysis, Investigation, Software, Writing – original draft. ZG: Formal analysis, Resources, Validation, Visualization, Writing – original draft. JZ: Conceptualization, Funding acquisition, Investigation, Methodology, Project administration, Supervision, Visualization, Writing – original draft, Writing – review & editing. DX: Conceptualization, Funding acquisition, Project administration, Supervision, Visualization, Writing – original draft, Writing – review & editing.
